# The Role of the Sense of Belonging During the Alarm Situation and Return to the New Normality of the 2020 Coronavirus Pandemic (COVID-19) in Spain

**DOI:** 10.1007/s12646-021-00612-z

**Published:** 2021-07-28

**Authors:** Jesús Saiz, Clara González-Sanguino, Berta Ausín, Miguel Ángel Castellanos, Ana Abad, María Salazar, Manuel Muñoz

**Affiliations:** 1 School of Psychology, Department of Social, Work and Differential Psychology, Chair Against Stigma Grupo 5-Complutense University of Madrid, Complutense University of Madrid, Mailbox 274, Somosaguas Campus, 28223 Pozuelo de Alarcón, Madrid, Spain; 2 Personality, Evaluation and Clinical Psychology Department, Chair Against Stigma Grupo 5-Complutense University of Madrid, Complutense University of Madrid, Madrid, Spain; 3 Psychobiology and Methodology in Behavioral Sciences Department, Chair Against Stigma Grupo 5-Complutense University of Madrid, Complutense University of Madrid, Madrid, Spain; 4 Chair Against Stigma Grupo 5-Complutense University of Madrid, Madrid, Spain

**Keywords:** COVID-19, Mental health, Quarantine, Sense of belonging, Social support

## Abstract

In this study we intend to understand the impact of the COVID-19 crisis and the subsequent stay-at-home orders, on the Spanish population's sense of belonging at three moments in time: at the beginning of the lockdown, after one month of lockdown and with the return to the “new normality”. A cross-sectional study was conducted through an online survey (N_0_ = 3480; N_1_ = 1041; N_2_ = 569). The sense of belonging was evaluated by means of four Likert-type items. These questions included membership in different groups: work/studies, friends, family and neighborhood or community. Sociodemographic and COVID-19-related data were collected. Additionally, mental health, spiritual well-being, loneliness, social support and discrimination were assessed. Descriptive analyses were carried out and linear regression models compiled. The sense of belonging increased significantly during confinement, dropping dramatically with the start of the return to the “new normality” process. The only variable that showed interaction with time and sense of belonging was discrimination. Work condition (not working providing the lowest sense of belonging scores), social support from friends and loneliness were the main predictors of the sense of belonging. The impact caused by the pandemic and the actions adopted during the first weeks regarding the sense of belonging is evident. It has been a key variable in dealing with COVID-19. Actions are now needed to increase our sense of belonging to face the post-epidemic crisis and avoid a greater impact in other areas.

## Introduction

The COVID-19 pandemic has had an unprecedented impact on our societies. Most countries had to declare a state of emergency, with physical distancing of the population being applied as the main measure. Spain was one of the countries hit the hardest by the pandemic when it reached Europe, leading the world in the number of infected people and people who died. At the beginning of July 2020, over 28,000 people had died and more than 250,000 people had been infected in Spain, making it the third country in Europe with the most cases (Health Ministry, Government of Spain, 2020).

The pandemic had triggered a social, economic and health crisis that has had a major impact on our mental health and the scientific community has made a great effort to clarify the mechanisms that might help us deal with this situation. Most research points to the emergence of symptoms of anxiety, depression, sleep problems and post-traumatic stress disorder (PTSD) in a significant percentage of the population (González-Sanguino et al., 2020; Mazza et al., 2020; Tanoue et al., 2020; Voitsidis et al., 2020; Wang et al., 2020a). In addition, others have argued that if our identities are defined in a substantial part by the groups to which we belong (Tajfel et al., 1979), our sense of self can be profoundly shaken when we are separated from these groups (Jetten et al., 2020). As a consequence, the social isolation that results from lockdown policies can lead to loneliness (Fortgang, 2021), with the need to maintain social connectedness (despite physical isolation) becoming a mandatory recommendation for mental health (Courtet et al., 2020).

As the Belongingness hypothesis defines (Baumeister et al., 1995, p.497) “human beings have a pervasive drive to form and maintain at least a minimum quantity of lasting, positive and significant interpersonal relationships”. In this way, this feeling can be essential when dealing with complicated situations or social and economic crises, as it is related to better psychological and social functioning (Hagerty et al., 1996). The need to belong or sense of belonging (SB) has even been found to provide protection in the presence of depression and suicidal thoughts (McCallum et al., 2010; McLaren et al., 2009). According to this, SB has been previously associated to mental health (Hagerty et al., 1992).

Complementarily, SB has been found closely related to well-being too (Ausín et al., 2017; Mellor et al., 2008). There are many approches to study well-being. Keyes et al. (2002) defined subjective well-being as “the evaluation of life in terms of satisfaction and balance between positive and negative affect” and psychological well-being (p.1007) as “the perception of engagement with existential challenges of life” (p.1007). Spiritual well-being has been described as a “dynamic and affective dimension of religion and spirituality that impacts the way that people experience, understand and live their lives” (Munoz et al., 2015, p.2). Spiritual well-being has been successfully used in clinical and non-clinical settings and has proved to be an important protective factor during COVID-19 pandemic (González-Sanguino et al., 2020).

Social support, discrimination and loneliness can be considered social connectedness variables very close related to SB. For instance, Hagerty et al. (1999) found that SB, loneliness and social support had a strong correlation when explaining depression; and Liu et al. (2014) probed that discrimination and SB explained loneliness.

In addition, Spain is a country where social relations play a role fundamental role, with strong social and family networks, where the SB and loyalty to the group plays a fundamental axis in our life. Moreover, it has a very high number of infections and deaths and the socioeconomic crisis caused by the COVID-19 has also been one of the most significant. This study is unique, since it informs us of our SB throughout the evolution of the months of the pandemic and its relationship with other variables. Knowing this information can be very useful for us to face the current second wave of the pandemic, as well as future similar crisis situations.

Considering these groups’ SB and relationships, the physical isolation stemming from the health emergency has had a great psychological impact on specific groups, such as young people and students (Becerra-García et al., 2020; Liu et al., 2020; Losada-Baltar et al., 2020; Moreira et al., 2020; Wang et al., 2020a), women (Alon et al., 2020; Song et al., 2020) and ethnic minorities and the self-employed (Platt et al., 2020).

It should be noted that despite the emergence of multiple studies that account for the psychological consequences of the pandemic (Huckins et al., 2020; Li et al., 2020; Wang et al., 2020b; Zhang et al., 2020), most are of a cross-sectional nature, with few longitudinal studies reporting how the crisis caused by the pandemic has affected our mental health over time. It is even more difficult to find data on this evolution that includes the “new normality,” once the countries have opened up and the impact on variables such as SB. In addition, considering the importance of social connectedness factors for well-being and mental health, it is necessary to explore the mechanisms by which the SB can be explained.

This longitudinal study aims to explain the impact of the crisis and lockdown situation on the population's SB, at three moments in time: at the beginning of the confinement, after a month and after two months, with the reopening and return to the “new normality.”

## Methods

### Procedure

In Spain, the state of emergency was declared on March 14, drastic isolation measures were applied to all citizens, including the total suspension of all work activity not considered essential from March 30 to April 12 and increasing physical isolation as much as possible. On May 4 the reopening process began and the restrictions were gradually lifted until they were completely removed on June 21, with the arrival of the “new normality.” A longitudinal study with 3 evaluations was carried out from March 21 to June 4. The use of longitudinal design is required because it can inform us of the SB throughout the evolution of the months of the pandemic and its relationship with other variables.

The first evaluation (T0) was carried out from March 21 to March 29, assessing the initial impact of the situation. The second evaluation (T1) was carried out from April 13 to 27, reflecting the evolution of the impact during the toughest moments of the confinement with the greatest impact at the socioeconomic level. The third and last evaluation (T2) took place from May 21 to June 4 and assessed the consequences of the lockdown and the process of lifting the restrictive measures.

The evaluations were carried out by means of an online survey (80 items, 10 min of approximate duration). The study received the approval of the Deontological Commission of the Faculty of Psychology of the Complutense University of Madrid (pr_2019_20_029) prior to its implementation. The signing of the informed consent and acceptance of the data protection laws were also included in the evaluation.

### Participants

Recruitment consisted of sending requests for participation to people belonging to databases of different institutions: students and workers in public organizations such as the Complutense University of Madrid and the academic Chair Against Stigma and private organizations such as the company Group 5. To increase the sample size as much as possible participants were asked to help with its dissemination by sending the survey through various social network channels (email, Twitter, distribution through WhatsApp lists, Facebook…) and on the website www.contraelestigma.com. A total of 3480 people participated in the first evaluation. For the subsequent evaluations, those people who had previously agreed to participate in the study were contacted by email; 1041 participants were recruited in the second data collection and 569 in the third. The inclusion criteria were: 1. Being over 18 years old; 2. Living in Spain during the state of health emergency caused by COVID-19; 3. Agreement to participate in the study.

The survey included a section with the consent form to participate in the study and acceptance of the data protection laws in regulation (EU) 2016/679 of the European Parliament and of the Council, of April 27, 2016, on the protection of personal data. The study was approved by the Deontological Commission of the Faculty of Psychology of the Complutense University of Madrid with reference “pr_2019_20_029."

### Instruments

#### Sociodemographic and COVID-19 Related Variables

The following variables are shown in Tables 1 and 2 and were collected through ad hoc questions: Age (subsequently grouped into clusters: 18–30, 31–59, 60–80); gender identity (women, men, others); civil status (single, married, divorced, separated, widower); educational level (elementary studies, high school, vocational training, university, postgraduate); profession (social-health, education, administration, commercial, other); work situation (working, unemployed, student, retired, others); work condition (work for others, self-employed, no work); economic situation (subjective perception from very bad to very good). The information regarding the pandemic was the following: suffering from symptoms (yes, no); existence or not of family members or close persons who were infected; living with an infected person; perception of the information received on the alarm situation (considers that he or she has sufficient information or is over-informed); employment during COVID-19 (obliged to go to his or her work center or telework).

**Table 1:** Sociodemographic variables in the three evaluations

Variables	T1 N (%)	T2 N (%)	T3 N (%)
*Gender*			
Men	860 (25)	202 (19)	104 (19)
Women	2584 (75)	841 (81)	453 (81)
*Age*			
18–29	1216 (35)	306 (29)	148 (27)
30–59	2035 (59)	670 (64)	364 (65)
> 60	200 (06)	69 (07)	46 (08)
*Civil Status*			
Single	1900 (55)	542 (52)	268 (48)
Married	1231 (36)	386 (37)	227 (41)
Divorced	214 (06)	82 (08)	42 (08)
Separated	67 (02)	28 (03)	17 (03)
Widow	39 (01)	7 (01)	4 (01)
*Education*			
Elementary	98 (03)	15 (01)	6 (01)
High school	599 (17)	149 (14)	69 (12)
Vocational training	439 (13)	125 (12)	68 (12)
University	1294 (37)	401 (38)	216 (39)
Posgraduate	1021 (30)	355 (34)	199 (36)
*Professional area*			
Administration	332 (10)	95 (09)	49 (09)
Commercial	208 (06)	55 (05)	29 (05)
Education	542 (16)	179 (17)	108 (19)
Social-health	1025 (30)	348 (33)	181 (32)
Other (security forces, lawyer…)	1344 (39)	368 (35)	191 (34)
*Work situation*			
Unemployed	283 (08)	92 (09)	54 (10)
Student	655 (19)	180 (17)	86 (15)
Retired	122 (04)	48 (05)	35 (06)
Other	212 (06)	120 (11)	70 (13)
Working	2173 (63)	604 (58)	312 (56)
*Work condition*			
Work for others	2200 (64)	686 (66)	367 (66)
Self-employed	396 (11)	114 (11)	62 (11)
No work	855 (25)	245 (23)	129 (23)
*Economic situation*			
Very bad-bad	348 (10)	111 (11)	58 (10)
Good-very Good	1975 (59)	621 (60)	359 (65)
Not good or bad	1042 (31)	304 (29)	137 (25)

**Table 2:** COVID-19 related variables in the three evaluations

Variables	T1 N (%)	T2 N (%)	T3 N (%)
*COVID-19 symptoms*			
No	2974 (86)	836 (80)	445 (80)
Yes	477 (14)	209 (20)	113 (20)
*COVID-19 diagnosis of a relative*			
No	2474 (72)	638 (61)	380 (68)
Yes	977 (28)	407 (39)	178 (32)
*Living with someone infected*			
No	3358 (97)	1016 (97)	550 (99)
Yes	93 (03)	29 (03)	8 (01)
*Information received*			
Not enough	614 (18)	184 (18)	96 (17)
Good	1983 (57)	594 (57)	326 (58)
Over-informed	854 (25)	267 (26)	136 (24)
*Employment during COVID-19*			
Not applicable	1398 (41)	427 (41)	233 (42)
Work on site	565 (16)	148 (14)	107 (19)
Work from home	1488 (43)	470 (45)	218 (39)

#### Social Connectedness Variables

- The SB to different groups was evaluated through four Likert-type items (1 much—4 nothing) employed in previous studies (Hernán-Montalbán et al., 2017). These questions included membership in: work/study groups, friends, family and neighborhood or community. Lower scores indicate lower membership. The scale´s consistency is low (α = 0.58), as each element reflects a very different group belonging.
- Social support was evaluated by the Multidimensional Scale of Perceived Social Support (EMAS) adapted to Spanish (Landeta et al., 2002; Zimet et al., 1988). The scale is made up of 12 Likert-type items with 7 possible responses (1 totally disagree to 7 totally agree), with the higher number being the more social support perceived. The EMAS explores three possible sources of perceived social support: family (4 items), friends (4 items) and relevant people (4 items) and also offers a full measure of social support. Cronbach’s α for the Spanish version is 0.89. These characteristics are what recommended the use of this test over others also validated in Spanish (e.g., Gallardo-Peralta et al., 2021).
- Loneliness was measured by the 3-item version of the UCLA Loneliness Scale (UCLA-3) in its Spanish version and self-applied (Russell, 1996; Velarde-Mayol et al., 2016). The three items, in Likert format with three response options (1 rarely, 2 sometimes, 3 often), address three dimensions of loneliness: relational connection, social connection and self-perceived isolation. Cronbach’s α for the Spanish version is 0.95.
- Discrimination was evaluated by means of the Intersectional Day-to-Day Discrimination Index (InDI-D) (Scheim et al., 2019), in its Spanish version. The translation was carried out by the authors of this study. This scale provides a measure of the intersectional discrimination that can be caused by different conditions: gender, ethnicity, mental health diagnosis and in this case, the presence of COVID-19 was also included. We used the main scale formed by 9 Likert-type items with four possible responses (1 never—4 often). The different questions evaluated the presence of intersectional discrimination from the beginning of the state of emergency caused by the coronavirus. The higher the score, the more discrimination suffered. For the Spanish version the scale´s consistency was adequate (α = 0.76).

#### Psychological Impact (mental health and spiritual well-being)

- Possible symptoms were evaluated with the following screening instruments: Patient Health Questionnaire 2 (PHQ-2), in its Spanish version (Diez-Quevedo et al., 2001; Kroenke et al., 2009). This brief self-report questionnaire addresses the frequency of symptoms of depression. The original scale presented a sensitivity of 0.9 and a specificity of 0.61 (Kroenke et al., 2009). Generalized Anxiety Disorder Scale-2 (GAD-2) in its Spanish version (Garcia-Campayo et al., 2014; Spitzer et al., 2006), which evaluates the presence of anxious symptoms. Both tests are made up of 2 Likert-type questions ranging from 0 never, to 3 every day. Higher scores indicate more symptoms. The sensitivity of the original test was 0.88; with a specificity of 0.61). Posttraumatic Stress Disorder Checklist (PCL-C) in Spanish (Lang et al., 2005). This questionnaire was used to detect post-traumatic symptoms. A reduced version of two Likert-type items was chosen, which asks about the presence of and how the person was affected by certain phenomena related to the traumatic experience. The answers range from 0 nothing to 4 extremely. The original version shows a sensitivity of 0.96 and a specificity of 0.58.
- Spiritual well-being was assessed using 4 items from the meaning/peace subscale of the Spanish version of the Functional Assessment of Chronic Illness Therapy Spiritual Well-Being (FACIT-Sp12) (Cella et al., 1998). The answers were Likert type from 0 (nothing) to 4 (a lot). Higher scores indicate greater spiritual well-being. For the meaning/peace subscale, Cronbach’s α was 0.88

### Analysis

To analyze the effect of longitudinal measures, linear mixed models were calculated for SB. Because the data contain missing values (participants who did not respond to successive surveys), the random effects were calculated as random slopes (without random intercepts) so that the models could be estimated. The results include the value of Nakagawa's Pseudo-R2 (marginal and conditional). The first considers exclusively the variances of the fixed component, while the second takes into account both the fixed and random effects. Post hoc comparisons were calculated using the estimated marginal means with Tukey adjustment. The analyses were performed using R (v3.5.6) with the lme4 and emmeans packages.

## Results

### Characteristics of the Sample

The sample in all the evaluations was made up of a high proportion of women (75, 81 and 81%), with a majority between 30 and 59 years of age (59, 64 and 65%) and mostly with single people (54%, 52% and 47%). In general, the respondents had university or postgraduate studies (67, 72 and 75%), with a job at the time of evaluation (63, 58 and 56%), working for others (63, 65 and 64%) and assessing their personal financial situation as good to very good (59, 60 and 65%).

Most people did not claim to have suffered from symptoms of COVID-19 (86, 80 and 80%). On the other hand, a significant proportion had a family member or close relative who had been infected by the virus (28, 39 and 32%). Finally, most people commented that they had had enough information during the pandemic (57, 57, 58%) and most of the sample had continued to work from home instead of going out to work (43, 45 and 39%).

The results across the three longitudinal assessments on the sociodemographic variables and the scores on the main scales can be seen in Tables 1 and 2.

### Longitudinal Changes in Sense of Belonging and Selected Variables

**Fig. 1:**
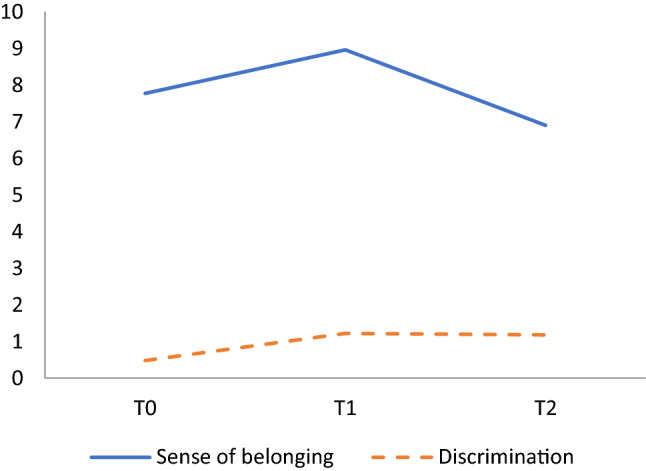
Results and trends for Sense of Belonging and Discrimination in the three evaluations

As shown in Fig. 1, in the scores related to SB, a significant increase is observed in the second evaluation (Z(T0-T1) = 17.51, *p* < 0.001). This decreases drastically from the second to the third (Z(T1-T2) = 21.11, *p* < 0.001) and even drops below the previous levels, with significant differences between the first and third evaluations (Z(T0-T2) = 10.25, *p* < 0.001).

The rest of the other variables did not seem to change over time, except Discrimination. This variable actually interacted with time and SB, increasing also in the second evaluation, albeit holding at the same level in the third evaluation. These results are shown in Table 3 and Fig. 1.

**Table 3:** Association between selected variables and Sense of Belonging over Time

Variables*	T0 (M, SD)	T1 (M, SD)	T2 (M, SD)	Time (*p* value)	SB x Time (*p* value)
SB**	7.77 (1.97)	8.96 (1.64)	6.90 (2.14)	0.001	
Social Support	51.74 (8.51)	51.08 (8.82)	51.03 (8.50)	0.079	0.077
SS-Family	17.34 (3.33)	16.81 (3.52)	16.84 (3.29)	0.305	0.336
SS-Friends	17.27 (3.37)	16.97 (3.28)	16.92 (3.26)	0.288	0.294
SS-Others	17.52 (3.41)	17.30 (3.60)	17.26 (3.53)	0.394	0.371
UCLA-3	4.55 (1.63)	4.53 (1.65)	4.30 (1.52)	0.151	0.254
InDI-D	0.48 (1.31)	1.22 (2.08)	1.18 (2.04)	0.009	0.001
PHQ-2	1.60 (1.51)	1.81 (1.43)	1.65 (1.40)	0.326	0.200
GAD-2	1.79 (1.63)	1.80 (1.57)	1.73 (1.51)	0.085	0.051
PCL-C	1.42 (1.84)	1.38 (1.81)	1.18 (1.70)	0.195	0.124
FACIT-SP	15.61 (3.29)	15.54 (3.33)	15.72 (3.27)	0.173	0.133

*SB* Sense of belonging.

*The association between all selected variables and SB is significant (*p* < .001).

**For SB only the difference over time is shown (Z [T2 < T0 < T1], *p* < 0.001)

### Regression Equations on Sense of Belonging

Although all the variables used showed a significant relationship with SB, the regression model showed how for SB only three variables appeared as the main predictors: Work condition [*F*(4,4283.3) = 200.57, *p* < 0.001], social support [*F*(1,4860.9) = 147.15, *p* < 0.001] and loneliness [*F*(1,4907.6) = 69.63, *p* < 0.001]. Specifically, the data showed that no working received the lowest scores on SB, high social support facilitated by friends increased SB and loneliness was negatively associated with SB.

The model explains 18.9% of the variance of the fixed effects, and the results and model developed are shown in Table 4.

**Table 4:** Results for Sense of Belonging

Fixed effects	Mean Sq	df1	df2	F	*p*
Time	2.19	1	1167.8	0.61	0.4335
Work condition	841.31	4	4283.3	200.57	< .0.001
SS-Friends	524.48	1	4860.9	147.15	< 0.001
UCLA-3	248.19	1	4907.6	69.63	< 0.001

Random effects			Pseudo-R^2^		
Time|id	0.041		Conditional	0.189	
Residual	3.564		Marginal	0.086	

*SS-Friends* Social Support, source Friends. Work condition includes work for others, self-employed, no work

## Discussion

The results show the evolution of the SB throughout the COVID-19 crisis and when restrictions began to be lifted. The observed trend shows how during confinement the SB scores increased significantly and then decreased dramatically with the arrival of reopening and the beginning of the “new normality.” Considering SB as a facilitator for health (Begen et al., 2015; Hagerty et al., 1992), this shows how during confinement to our homes and when the crisis situation was more acute and complicated, reaching out and relying on our communities was perhaps a strategy for coping with the situation. During the months of lockdown, several social changes could have perhaps increased the SB. The spokesperson for the Health Alert and Emergency Coordination Centre of the Ministry of Health appeared in daily press conferences. There were weekly appearances by the President of the Government, multiple television announcements providing information about the steps being taken to combat COVID-19 and announcements calling for unity. Citizens applauded from their balconies every day to thank the work of the health workers. They played the song “I will resist” constantly. Spontaneous community support networks sprang up, as well as the multiple posters hung in the windows painted with rainbows with the message “*Everything will be all right” (or “todo irá bien” in Spain and “tutto andrà bene”* in Italy). However, as the country went from the acute crisis and began to return to the “new normality,” the SB scores dropped dramatically and even fell below the levels found in the first assessment. This may have been due to the climate of political tension in the country and pressure from social movements linked to extreme political parties that denied the existence of the crisis and the risks. Additionally, demonstrations were organized in the streets, the economic situation became worse (Nicola et al., 2020) and there were fewer and fewer of the actions mentioned above that initially seemed to unite the country in the most difficult moments (i.e., applause on the balconies). On the other hand, it must be taken into account that the sample studied was made up of a majority of women and people between the ages of 30 and 59 years, single and with university or postgraduate studies who were working at the time of the interview with a good evaluation of their economic level. It should also be noted that in relation to the COVID-19 most people had not had any symptoms or affected people close to them, feeling that the information they had received about the pandemic in general was good. In this sense it should be noted that the results found could have been different if the sample had had different sociodemographic characteristics, such as groups with a worse socioeconomic situation where perhaps the SB could have increased to a lesser extent as they were less supported by the situation. Or people with symptoms or affected family members where perhaps there would have been greater isolation or even discrimination due to the illness, affecting their SB.

All of the selected variables introduced showed significant associations with SB. However, the only variable that interacts over time was intersectional Discrimination. This opens the door to several possible interpretations that may need further research and discussion. Firstly, while SB decreases, discrimination maintains high. In the second wave the turning point occurs. This happens once we feel familiar with the situation we begin to withdraw from our SB and avoid people who belong to groups that can be potentially contagious. Specifically, at the beginning we all belong to the extended group of those who want to beat the virus and then we begin to differentiate between healthy people and those who could infect us (regardless of whether they are health workers, supermarket cashiers, truckers and so on). Secondly, this might also suggest that people feel more discriminated against during the hardest part of the pandemic and the more the person feels discriminated the less they feel they belong. Discrimination against certain health conditions is common, having also been recorded in previous pandemics, where infected or suspected persons, as well as health care workers, suffered from greater discrimination (Desclaux et al., 2017; DiGiovanni et al., 2004; Lee et al., 2005; Pellecchia et al., 2015; Reynolds et al., 2008; Wilken et al., 2017).

On the other hand, three variables explained the role of SB during the alarm situation and the return to the “new normality”: the work conditions (with “not working” recording the lowest scores on SB), social support facilitated by friends and loneliness. Wilcock (2006) argues that psychological and social well-being, as essential aspects in the current concept of health, are linked to the capacity of the human being to develop socially valued occupations. Thus, participation in occupations is essential for the human being. In her postulate (Wilcock, 2006), the author defends what she calls the "occupational nature of people" at the same level as being social by nature. According to the American Occupational Therapy Association (AOTA, 2020), in order to provide a sense of well-being, there must be a balance between the occupational areas of self-care, work and leisure. Indeed, as Moruno-Miralles (2020) has stated, work can be considered as a significant occupation that allows the person to fulfill the social demands of his community of belonging and constitutes a way of giving meaning to existence and building one’s personal, cultural and social identity. In our sample, work was clearly associated with SB and not having work affected SB negatively. This is consistent with McClure et al. (2008), who already described the importance of belonging at work.

Considering that “social support refers to a social network’s provision of psychological and material resources intended to benefit an individual’s ability to cope with stress” (Cohen, 2004, p.676), its association with SB reinforces the necessity to maintain social connections during lockdown periods to maintain well-being (Brooks et al., 2020), or even to prevent suicide (Courtet et al., 2020).

Finally, perceived loneliness was an important variable in the results. The experience of loneliness has been conceptualized as the cognitions and attributions that arise when an individual perceives a discrepancy between their needed and existing social relationships (Perlman, 2004). We find perceived loneliness to be the third variable to explain SB for our sample, underlining the importance of this variable in the relationship of building our SB. The major role of loneliness in relation with COVID-19 consequences is in line with some studies that have already demonstrated the association between loneliness and anxiety, depression and PSTD in the current confinement situation (González-Sanguino et al., 2020; Santini et al., 2020).

Limitations in the study include the loss of participants over time, especially in the third assessment, which may be a sign of a return to normality and loss of interest in the phenomenon. Additionally, our sampling procedure does not ensure that the sample of the population is representative and it under represents certain groups such as men and the elderly. Finally, although the scale to evaluate SB was used successfully here and in previous studies, the scale´s consistency is low, so it would be necessary to use more accurate instruments in future explorations.

## Conclusion

In this era of lockdown, the demands on SB have been for the most part those related to productive occupational areas, such as the roles of worker, student, and childcare. In many cases, the confinement stage has led us to participate in an excessive number of tasks and activities, with the consequent difficulty of spreading them out evenly and many had nothing to do with enjoyment and pleasure. Begen et al. (2015) found that acting to enhance belonging through inclusion resulted in adaptive physiological and psychological outcomes. Thus, to cope better with this crisis, it seems advisable that SB should be reinforced, possibly by finding a better balance between our other occupations, including the time we spend at work and with our friends and by combating the feelings of loneliness.
